# Structure of Ddn, the Deazaflavin-Dependent Nitroreductase from *Mycobacterium tuberculosis* Involved in Bioreductive Activation of PA-824

**DOI:** 10.1016/j.str.2011.11.001

**Published:** 2012-01-11

**Authors:** Susan E. Cellitti, Jennifer Shaffer, David H. Jones, Tathagata Mukherjee, Meera Gurumurthy, Badry Bursulaya, Helena I. Boshoff, Inhee Choi, Amit Nayyar, Yong Sok Lee, Joseph Cherian, Pornwaratt Niyomrattanakit, Thomas Dick, Ujjini H. Manjunatha, Clifton E. Barry, Glen Spraggon, Bernhard H. Geierstanger

**Affiliations:** 1 Genomics Institute of the Novartis Research Foundation, 10675 John Jay Hopkins Drive, San Diego, CA 92121-1125, USA; 2 Tuberculosis Research Section, National Institute of Allergy and Infectious Diseases, National Institutes of Health, Bethesda, MD 20892, USA; 3 Novartis Institute for Tropical Diseases, 138670 Singapore; 4 Center for Molecular Modeling, Center for Information Technology, National Institutes of Health, Bethesda, MD 20892, USA

## Abstract

Tuberculosis continues to be a global health threat, making bicyclic nitroimidazoles an important new class of therapeutics. A deazaflavin-dependent nitroreductase (Ddn) from *Mycobacterium tuberculosis* catalyzes the reduction of nitroimidazoles such as PA-824, resulting in intracellular release of lethal reactive nitrogen species. The N-terminal 30 residues of Ddn are functionally important but are flexible or access multiple conformations, preventing structural characterization of the full-length, enzymatically active enzyme. Several structures were determined of a truncated, inactive Ddn protein core with and without bound F_420_ deazaflavin coenzyme as well as of a catalytically competent homolog from *Nocardia farcinica*. Mutagenesis studies based on these structures identified residues important for binding of F_420_ and PA-824. The proposed orientation of the tail of PA-824 toward the N terminus of Ddn is consistent with current structure-activity relationship data.

## Graphical abstract

**Graphical Abstract:**

**Figure:**
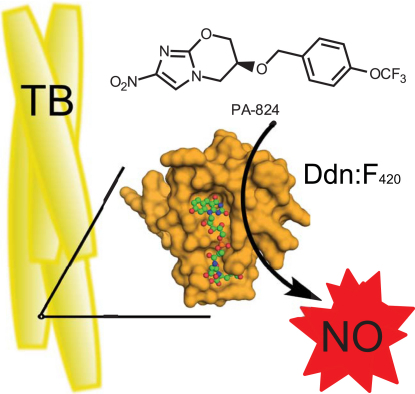

**Highlights:**

► Crystal structures of Ddn, the reductase for antitubercular drug PA-824 ► Active site residues and F_420_-binding mode described ► The N terminus is important for PA-824 binding but could not be fully characterized ► An active homolog from *Nocardia farcinica* was identified and studied

## Introduction

It is estimated that one-third of the world's population is infected with *Mycobacterium tuberculosis* (*MTB*) (Dye et al., 1999). In 2008, 11.1 million reported cases of active tuberculosis (TB) resulted in 1.3 million deaths worldwide (World Health Organization, 2011). *MTB* is able to evade the human immune response in part by triggering formation of insulating hypoxic granulomas following infection of pulmonary macrophages. Bacilli within this environment have adapted to slowly replicate and respire, making them tolerant of many drugs. This resistant state is thought to contribute to the prolonged combination chemotherapy required to cure patients (Barry et al., 2009; Boshoff and Barry, 2005). Lack of compliance with treatments lasting up to 9 months contributes to the emergence of resistant strains (Sharma and Mohan, 2006). The spread of multidrug-resistant strains and the rise of TB as a secondary infection in AIDS patients (Corbett et al., 2003) have increased the urgency of identifying new drugs to provide more practical treatments.

**Figure 1:**
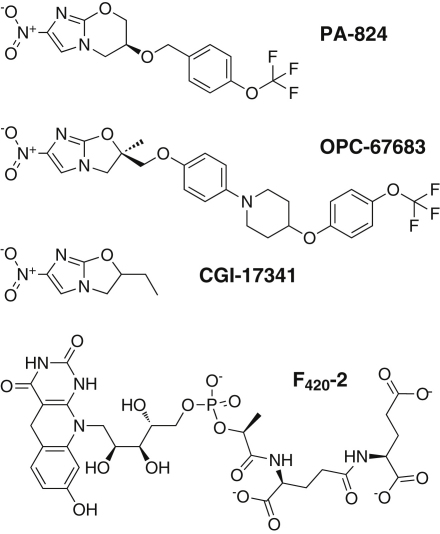
Structures of Bicyclic 4-Nitroimidazoles and of the F_420_ Coenzyme Purified F_420_ used in this work is a mixture with primarily four, five, or six glutamate residues. For simplicity, only the two residues that could be refined during costructure determination are shown.

Recently, bicyclic 4-nitroimidazoles have emerged as a new class of drug candidates against TB (Stover et al., 2000). PA-824 and OPC-67683 (Figure 1) are currently in phase II clinical trials and have shown efficacy against aerobically replicating bacilli, nonreplicating bacilli within hypoxic granulomas, and multidrug-resistant strains in preclinical animal models (Hu et al., 2008; Hurdle et al., 2008; Matsumoto et al., 2006; Stover et al., 2000; Tyagi et al., 2005). Both compounds not only disrupt mycolic acid synthesis, important for cell wall formation under aerobic conditions (Matsumoto et al., 2006; Stover et al., 2000), but also show considerably more complex cellular effects, particularly under anaerobic conditions where there are clear signs that respiratory poisoning is responsible for bactericidal activity (Manjunatha et al., 2009).

PA-824 and OPC-67683 are prodrugs that appear to undergo similar reduction reactions (Anderson et al., 2008; Maroz et al., 2010; Matsumoto et al., 2006; Singh et al., 2008; Thompson et al., 2009). Characterization of in vitro selected PA-824-resistant mutants identified lesions in genes coding for enzymes involved in F_420_ coenzyme (Figure 1) biosynthesis, F_420_-dependent glucose-6-phosphate dehydrogenase (FGD1), and Rv3547, a protein of unknown cellular function. Strains with disruptions in *fgd1* and *Rv3547* could be made sensitive to PA-824 by complementation with intact copies of the respective genes (Manjunatha et al., 2006a; Stover et al., 2000). Both FGD1 and Rv3547 are F_420_-dependent enzymes, but only Rv3547 is capable of reducing PA-824 directly (Manjunatha et al., 2006a), whereas FGD1 recycles the coenzyme to the reduced form. Because of its enzymatic activity, Rv3547 was named a deazaflavin-dependent nitroreductase (Ddn). The F_420_-dependent reduction of PA-824 has been shown to produce a mixture of three stable metabolites as well as a release of reactive nitrogen species (Singh et al., 2008). Of the three metabolites, the levels of a des-nitro species correlate best with nitric oxide (NO) release and anaerobic bactericidal activity. The role of NO as primary toxin is supported by previous suggestions that NO production by macrophages controls TB in murine models and in human macrophage cultures (MacMicking et al., 1995; Rich et al., 1997).

*MTB* proteomics studies have suggested that Ddn may be a peripheral membrane protein (Sinha et al., 2002, 2005), but its biological substrate and function remain unknown, and there is low sequence homology to any protein of known cellular function (Singh et al., 2008). Homologs do exist in several species, mostly within Actinobacteria, although not in *M. leprae*, the etiologic agent of human leprosy, which is resistant to PA-824 (Manjunatha et al., 2006b). The F_420_ coenzyme is only present in select groups of archaea and bacteria and is an obligate two-electron (hydride) donor with a more negative redox potential than nicotinamide coenzymes (Bair et al., 2001; Graham et al., 2003; Jacobson and Walsh, 1984).

In order to better understand the mechanism of activation of PA-824, we determined the structure of a truncated version of Ddn in the presence and absence of the F_420_ coenzyme. Comparison of these structures and NMR observations reveals regions of the protein that are conformationally flexible and undergo changes upon cofactor binding. We have also solved the structures of two full-length homologs from *Nocardia farcinica*, one of which is catalytically competent for PA-824 reduction in vitro. Structural and mutational studies with Ddn and the active homolog identified key residues in the active site of this enzyme family. The data presented in this study provide structural insights into the Ddn-catalyzed activation of bicyclic 4-nitroimidazole prodrugs, a process key to the function of this important class of antitubercular clinical candidates.

## Results

### Design and Construction of Soluble Forms of Ddn and Homologous Proteins

Currently, a maltose-binding protein (MBP) fusion (Kim et al., 2009b; Singh et al., 2008) is the only full-length wild-type Ddn construct that can be expressed as soluble protein in *Escherichia coli*. This MBP fusion protein activates PA-824 in vitro but is highly aggregated, as determined by size-exclusion chromatography. Untagged Ddn produced by proteolytic removal of MBP is also highly aggregated. Proteins that exist as soluble aggregates rarely produce crystals (Klock et al., 2008) and exhibit line-broadened NMR spectra, making them difficult targets for structural biology. Indeed, despite significant efforts, we have not obtained any suitable crystals or NMR spectra for full-length Ddn.

**Figure 2:**
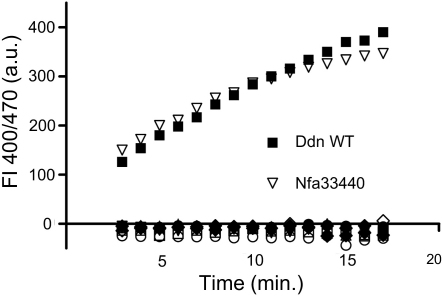
Activity of Ddn Constructs and Homologs The activities of several Ddn constructs were tested to identify those that could reduce PA-824, measured by an increase of fluorescence intensity (FI) of oxidized F_420_. None of the soluble, nonaggregated monomeric truncations or mutants showed activity (data plotted for F16A-F17D-W20D-I24A-W27D-M28A [white diamonds]; D15E-F16A-F17D-W20D-I24A-W27D [white circles]; F16A-F17D-W20D-I24A-W27E [white squares]; NΔ30 [black circles]; NΔ33 [black up triangles]; Δ14-22 [black down triangles]; Δ14-30 [black diamonds]). Homologous proteins for which structures were determined were also tested. Only nfa33440 showed activity toward PA-824. Assay conditions: 30–50 nM enzyme, 30 μM PA-824, 20 μM F_420_H_2_, 0.2 M Tris-HCl (pH 8.0), 0.01% Triton X-100, 25°C. Similar results were obtained with 300 nM enzyme (data not shown). See Figures S1–S3.

To identify proteins more suitable for structural biology, we tested a series of 17 truncation mutants (see Table S1 available online). Deletion of 26–50 residues from the N terminus showed high levels of soluble protein expression, and removal of 30, 33, or 40 residues produced nonaggregated monomeric protein (Figure S1A). Well-dispersed signals for 113 of 114 nonproline residues were observed by ^1^H-^15^N-HSQC NMR spectroscopy for uniformly ^15^N-labeled NΔ30 Ddn protein, suggesting a well-defined three-dimensional structure (Figure S1B). This sample also showed significant chemical shift perturbation upon addition of oxidized or reduced coenzyme F_420_, demonstrating tight binding of the coenzyme and suggesting that this construct adopts a physiologically relevant form (Figure S1B). None of the N-terminally truncated soluble constructs were catalytically competent as assessed by monitoring oxidation of F_420_ in the presence of PA-824 (Figure 2), suggesting that the N terminus is important for substrate binding or catalysis.

The N terminus of Ddn is predicted to contain an amphipathic α helix at residues 13–28 with a highly hydrophobic side (Figure S2) that may be causing aggregation of full-length Ddn. Consequently, we made a series of 36 mutants designed to disrupt this hydrophobic surface, including combinations of up to six point mutations (Table S1). Some combinations of three or more mutations resulted in soluble monomeric protein but did not show additional peaks in ^1^H-^15^N-HSQC spectra compared to the truncation mutants (Figure S3A), indicating that the N terminus in these mutants undergoes conformational exchange processes in the milli- to microsecond timescale. Other mutations designed to stabilize the hypothetical helix through salt bridges also resulted in monomeric proteins, but chemical shift analysis of HSQC spectra (Figure S3B) suggested that the mutated N termini did not interact with the core of the protein. All monomeric full-length mutant constructs have so far failed to produce diffracting protein crystals despite extensive screening of conditions in the presence and absence of coenzyme and PA-824. In addition, none of the mutant monomeric full-length proteins showed activity toward PA-824 even at high enzyme concentration (300 nM).

In parallel, we expressed several Ddn homologs that were chosen to have a range of lower predicted isoelectric points, a trait that has been shown to enhance crystallization in the context of structural genomics (Slabinski et al., 2007). These proteins were tested for activity and put into crystallization trials to determine protein structures that could add to our understanding of the full-length Ddn enzyme for which only the core structure could be determined.

### Ddn Structure Determination

**Figure 3:**
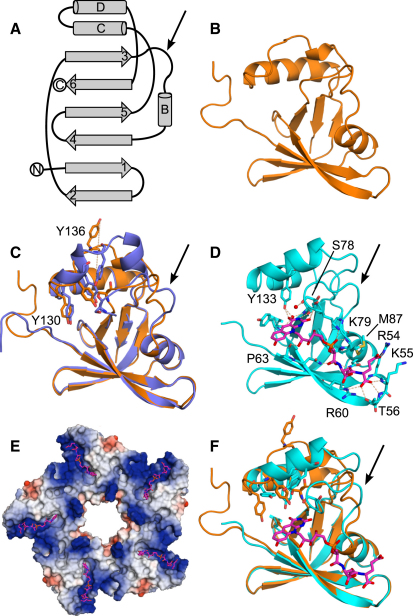
Ddn Structures with and without Coenzyme F_420_ (A) Topology of split barrel-like fold oriented to the models. Arrows point to the post-β3 loop discussed in the text for orientation throughout the figure. (B) Cartoon of Apo-1 structure showing split barrel-like fold. (C) Cartoon of one monomer from Apo-1 (orange) aligned with one monomer from Apo-2 (purple) and showing Y130, Y133, Y136, and S78 for comparison. Y130 and Y136 are labeled. (D) Cartoon of one monomer from the Holo-1 structure with F_420_ (magenta). The post-β3 loop (arrow) is repositioned to create the pocket for binding of the deazaflavin ring system. Key waters are shown as red spheres. (E) Surface rendering of the pentameric structure from Holo-1 shows a complementary groove with positive (blue) charges near the negatively charged tail of F_420_. A small pocket is formed near the flavin ring system but is not enclosed by the protein monomers or pentamers. Although the biological relevance is unclear, the pentamer provides room for the missing N termini (center of ring or packed on the front face). The front face also packs against a second pentamer in both crystal forms, occluding the active sites or potentially allowing crosstalk between active sites. (F) Cartoon of one monomer from Apo-1 (orange) aligned to one monomer from Holo-1 (cyan; F_420_ in magenta) and showing Y130, Y133, Y136, and S78 for comparison. See Table S2 for NMR characterization of NΔ30 Ddn with and without coenzyme.

An initial Ddn crystal structure was solved by single-wavelength anomalous dispersion (SAD) phasing of a 1.75 Å data set from a crystal belonging to space group C222_1_ of the NΔ33 construct with selenomethionine incorporated (Apo-0 in Table 1). As expected (Manjunatha et al., 2006a; Taylor et al., 2010), the backbone trace shows high similarity to proteins in the split barrel-like family of flavin-binding proteins (Figure 3A). The initial structure was then used to solve the structure of the longer NΔ30 construct by molecular replacement based on a 1.55 Å data set (Apo-1 in Table 1; Figure 3B) and of a 1.85 Å resolution NΔ33 crystal (P3_2_21 space group; Apo-2 in Table 1). All but the first five amino-terminal residues could be built into the electron density for both crystal forms (36–151 and 39–151, respectively). The asymmetric units (ASUs) for the Apo-1 and Apo-2 crystals both contain single molecules with similar structures (root-mean-square deviation [rmsd] is 0.96 Å). This alignment omits 14 of 113 shared Cα atoms because of conformational differences (Figure 3C) in the same regions that differ upon coenzyme binding as discussed below.

Cocrystals of the NΔ40 construct with oxidized F_420_ were also obtained in two space groups and were solved by molecular replacement (Holo-1 in C2 at 2.1 Å resolution and Holo-2 in P2_1_ at 1.8 Å resolution, with an rmsd for one chain of 0.3 Å over 109 aligned Cα atoms; see Table 1). The data from both crystals yielded clear difference electron density for the F_420_ coenzyme, with two glutamate residues in the tail that could be easily positioned (Figure 3D). Although the F_420_ preparation included larger polyglutamate tail species, only two glutamates could be visualized (F_420_-2), consistent with the structure of FGD1 crystallized with a similar preparation (Bashiri et al., 2008) and the recent observation that F_420_-2 and F_420_-5 bind with similar affinity to Ddn (Gurumurthy et al., 2012). The crystal packing combined with crystallographic symmetry operations reveals the same decameric ring structure in both structures (Figure 3E), with five monomers in the ASU of Holo-1 and ten in Holo-2 (two rings per ASU). Although the decamer structure includes extensive intersubunit packing, its relevance to the native quaternary structure and function remains unknown because of the difficulties of studying the full-length enzyme.

### Analysis of Ddn Structures and Conformational Changes upon Binding to F_420_

The split barrel-like fold is characterized by a central six-stranded antiparallel β barrel in a Greek key topology with four α helices: A at the N terminus (the putative helix removed for Ddn structure determination), B after an extended loop between strands β3 and β4, and C and D between strands β5 and β6 (Figure 3A). In Ddn, strands β2, β1, β4, and β5 form the larger sheet at the base of the molecule, whereas strands β2 and β5 are elongated to also interact with the shorter strands β3 and β6 in the middle of the molecule (Figure 3B).

**Figure 4:**
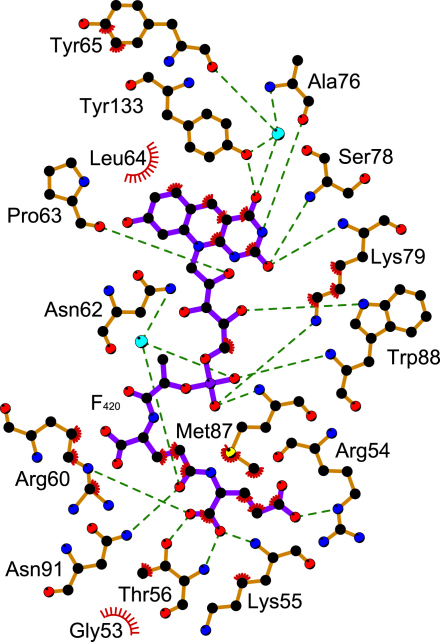
Analysis of the Interactions between Ddn and F_420_ One monomer from the Holo-1 structure (chains E and J) was input to LIGPLOT (Wallace et al., 1995) to exemplify the extensive hydrogen-bond and hydrophobic interactions between protein and cofactor. Bonds for the protein are colored brown; F_420_ bonds are purple (black, carbon; blue, nitrogen; red, oxygen; yellow, sulfur; purple, phosphorus). H bonds (3.2 Å cutoff) are indicated with green dashed lines; hydrophobic contacts are indicated with red dashes.

In structures Holo-1 and Holo-2, F_420_ binds to a groove on the surface of Ddn (Figures 3D and 3E) through a series of hydrogen bonds and several salt bridges between positive charges on Ddn and the negatively charged phosphate and tail of the coenzyme (Figure 4). The last visible glutamate of the F_420_ tail lies near the β1-β2 hairpin within hydrogen-bonding range of the side chains of R54, T56, and R60 as well as the main chain of K55 and T56. The more proximal glutamate H bonds with the side chain of N91 from helix B. The phosphate of F_420_ sits near the complementary positive charge of K79 and H bonds to the amides of M87 and W88 from the loop after strand β3. The ribityl moiety is stabilized by H bonds to the side chain of W88 (loop) and the main-chain carbonyl of P63 (strand β2). The deazaflavin ring system is stabilized by H bonds with the main chain of A76 and K79 and the side chain of Y133 (helix D). Additionally, there are waters in both costructures that mediate interactions between protein and coenzyme. The sum of these interactions results in the *Re* face of F_420_ being presented for reaction with PA-824.

The largest conformational changes between the apo- and F_420_-bound structures occur near the deazaflavin ring (Figure 3F). The loop following strand β3 (residues 76–85) shifts away from the binding site in the presence of coenzyme in order to form the direct contacts to the phosphate and deazaflavin heterocycle outlined above, with the Cα of S78 moving approximately 4 Å in Holo-1 versus Apo-1 (Figures 3D and 3E). In Holo-1 and Holo-2, this loop packs against α helix D (residues 131–139) and generally exhibits lower mobility, indicated by a drop in atomic displacement parameters (ADPs) relative to the apo structures. In Apo-1 and Apo-2, α helix D is highly disrupted, with repositioning of residues 129–144 relative to the holo-enzyme structures complementing movement of the post-β3 loop (Figure 3C). In Apo-1, the side chain of Y136 is flipped away from the F_420_ binding site, with the oxygen atom moving 19 Å relative to the Holo-1 structure. In Apo-2, this helix stops at Y136 and instead a helix forms upstream from residues 139–143, and residues 136–143 display higher ADPs. NMR data also suggest that the post-β3 loop and helix D are dynamic. Although 98% of the ^1^H-^15^N peaks for NΔ30 apo-Ddn have been assigned as part of this study, the spectra of the protein with F_420_ have proven less tractable, with the longest unassigned segments at residues 75–88 (post-β3 loop) and 122–148 (α helices C and D, and β6) and a shorter segment at 62–66 (β2) (Table S2). Approximately 75% of the peaks associated with these regions were missing or were too broad to be used for assignment. These data all suggest that portions of the protein are conformationally flexible, which may be functionally relevant, as exchange of the coenzyme would be required to complete each catalytic cycle.

### Structures of Ddn Homologs

**Figure 5:**
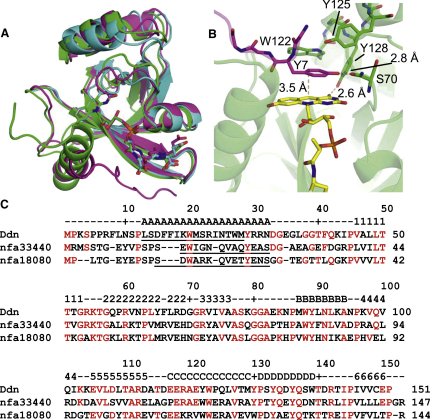
Structures of Proteins Homologous to Ddn (A) Superposition of Holo-1 (blue), nfa33440 (red), and nfa18080 (green) (see rmsd values in the text). The structures are shown aligned on coenzyme F_420_, which is represented by strong electron density in the same pose in all three data sets with variation mainly in the glutamate tail. (B) Detail of nfa18080 residue Y7 from one symmetry mate positioned in the active site of the neighboring monomer in the crystal. The tyrosine side chain interacts with the main chain of S70 (S78 in Ddn) and is positioned within 4 Å of the *Re* face of the flavin ring (measurement shown to C5). Residues W122, Y125, and Y128 (Ddn Y130, Y133, Y136) are also shown for reference. (C) Sequence alignment denoting identity (red) to Ddn from *MTB*. Secondary structure is conserved among the three holo-enzyme structures and is identified with reference to the topology map in Figure 3A (numbered β strands; lettered α helices) with the exception of α helix A, which is underlined (predicted for Ddn; experimental for homologs). See also Figure S4 for NMR characterization of nfa33440.

In order to characterize a full-length enzyme including the N terminus, we also determined the structure of nfa33440, a homolog from *N. farcinica* sharing 42% sequence identity to Ddn and that was active in the PA-824 in vitro assay (Figure 2). The structure of this protein was solved by molecular replacement using the Ddn structure. Although an initial apo structure lacked density for the first 37 amino acids, a cocrystal of nfa33440 with F_420_ confirmed that the overall structure and coenzyme binding are very similar to that of Ddn (Figure 5A; rmsd is 0.95 Å over 109 aligned Cα atoms to Holo-1). The N terminus of nfa33440 is represented in the electron density starting at residue 6, showing an extended sequence packed against the core of the protein followed by a short α helix flanking the F_420_ binding site. ^1^H-^15^N-HSQC NMR spectra were collected for nfa33440 alone, in the presence of F_420_, and with F_420_ and PA-824 (Figure S4). The spectrum of apo-nfa33440 includes approximately 107 ^1^H-^15^N peaks out of an expected 135. Although interpretation is uncertain without assigning the spectrum, these data, along with the lack of density for the N terminus in the apo-nfa33440 crystal, suggest that nfa33440 in solution consists of a structured core with the N terminus undergoing conformational exchange processes similar to the Ddn mutants. Addition of F_420_ to nfa33440 resulted in a spectrum with approximately 90 peaks. This is again similar to NΔ30 Ddn, in which parts of the active site appear to undergo conformational exchange in the presence of coenzyme. Addition of excess PA-824 to nfa33440 resulted in chemical shift perturbation of a few of the weaker peaks (Figure S4), supporting the hypothesis that it is residues in the active site that are undergoing dynamic processes.

The full-length structure of a second, inactive homolog, nfa18080 from *N. farcinica*, was solved by X-ray crystallography in the presence of F_420_ (rmsd to Holo-1 is 1.1 Å over 109 aligned Cα atoms). The protein core, short N-terminal helix, and coenzyme binding site are conserved for nfa18080 relative to nfa33440. Electron density for the N terminus of nfa18080 starts at residue 5 and approaches the active site of a neighboring monomer in the crystal. The side chain of Y7 of one monomer stacks on top of the exposed *Re* face of F_420_ (Figure 5B) of another monomer at the location where one would expect the enzyme substrate to bind (Fraaije and Mattevi, 2000). The homolog structures provide insight into possible orientation of some conserved N-terminal residues in relation to the active site. However, because of low sequence homology (Figure 5C; about 20% identity to Ddn residues 1–30) and flexibility of the N-terminal helix, these structures do not provide a reliable model for the missing Ddn N terminus. Parts of the active sites of Ddn therefore cannot be defined structurally, limiting the utility of these structures for elucidating the mechanism of NO generation from the PA-824 prodrug.

### Mutagenesis Studies and the Putative Binding Mode of PA-824 to Ddn

**Figure 6:**
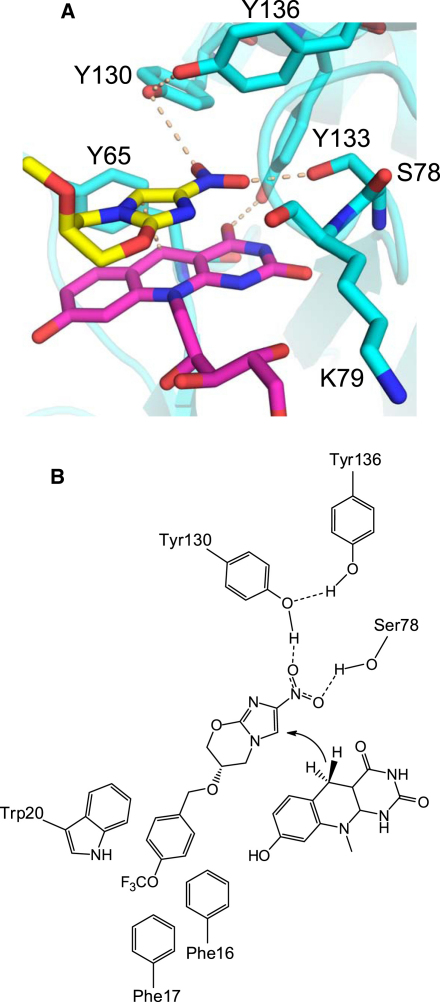
Putative Binding Mode of PA-824 (A) The 2-nitro-6,7-dihydro-5*H*-imidazo[2,1-b][1,3]oxazine core of PA-824 (yellow) was modeled into the Holo-1 structure near the F_420_ coenzyme. (B) Schematic showing residues thought to be important for PA-824 binding and catalysis. F16, F17, and W20 were not present in the X-ray structures of Ddn. Based on the mutation data, these residues are included to depict their potential interactions with the hydrophobic tail group of PA-824.

Attempts to determine the structure of a ternary complex of Ddn or a homolog with F_420_ and PA-824 or several analogs, by cocrystallization or soaking, were unsuccessful. Modeling was used to visualize the binding mode of PA-824 (Figure 6A), using the Holo-1 structure with F_420_ and placing the imidazole group of PA-824 at an appropriate distance from C5 of the flavin ring for hydride transfer (based on observations for other flavin-binding proteins; Fraaije and Mattevi, 2000). In the lowest-energy model, the nitroimidazole group of PA-824 is located near the *Re* face of F_420_, with the nitro group H bonded with the side chains of S78, Y130, and Y136 (Figure 6). Notably, S78 in Ddn and S72 in nfa33440 are bound to a water modeled near some chains of the crystal structures (shown for Holo-1, chain E in Figure 3D), which may mimic binding to one oxygen atom of the nitro group of PA-824. To test this hypothesis, we generated single point mutants in the full-length MBP-Ddn fusion protein as well as in nfa33440 and determined their enzymatic activity toward PA-824 at saturating concentrations of F_420_ (Table 2). Mutation of S78 (nfa33440 S72) resulted in loss of activity, as did perturbation of nearby residues likely to help position S78 (Ddn R142, I144; nfa33440 R136, I138). The proposed role of the Ddn Y130 (nfa33440 Y124) hydroxyl for substrate binding and orientation was also substantiated because mutation of Tyr to Phe significantly lowered catalytic competence in either enzyme. The activity of Ddn Y136 (nfa33440 Y130) was not significantly compromised by mutation to Phe, but was by mutation to Leu or Val. These data suggest that Y136 may not be directly involved in hydrogen bonding or proton relay but instead may contribute to aligning other aromatic residues in the active site.

Mutation of Y133 (nfa33440 Y127) resulted in reduced enzymatic activity (Table 2), suggesting a critical role for this residue in anchoring the deazaflavin ring of the coenzyme in the active site (Figure 4). Likewise, Y65, A76, and K79 are all likely to play a role in stabilizing the F_420_ complex. They uniformly resulted in loss of enzymatic activity. Among the equivalent nfa33440 mutants, decreased F_420_ affinity (Table S3) was observed for M59A (Ddn Y65), as assessed qualitatively by NMR. In the case of the nfa33440 mutants, we were able to confirm by NMR that mutations of these key residues did not result in large structural changes (Figure S5) or loss of affinity for F_420_ binding (M59A being the exception; measurable lower exchange rate) (Table S3). However, S72V, R136A, and I138A exhibited altered affinity for PA-824 (Table S4; Figure S6).

### Mutagenesis and Characterization of the N Terminus of Ddn

**Figure 7:**
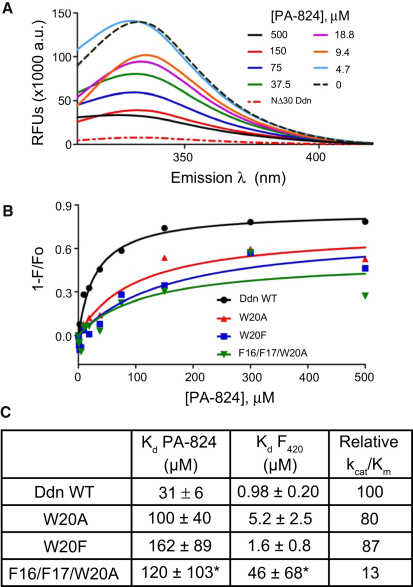
Binding of PA-824 to Ddn and Its Mutants Studied by Tryptophan Fluorescence Spectroscopy (A) Emission spectra in relative fluorescence units (RFUs) of Ddn in the presence of varying concentrations of PA-824 (0–500 μM) were recorded upon excitation at 280 nm. The intrinsic spectrum of the NΔ30 Ddn construct is also shown. For this experiment, untagged WT Ddn protein was used. (B) Titration of PA-824 in saturation binding analysis with the MBP fusion of Ddn WT and mutants monitored at 330 nm. The magnitude of fluorescence differences (1 − F/F_0_) was used for K_d_ calculation, as described in Experimental Procedures. (C) Values determined for MBP-Ddn mutants. Asterisks indicate the triple mutant which, even at saturating concentration of PA-824, showed less than 40% quenching, unlike WT MBP-Ddn, for which 80% quenching was observed. See Figure S8 and Table S5 for CD data on MBP-Ddn mutant proteins.

The loss of PA-824 activity in all truncated constructs suggested the importance of the N terminus, and studies of the intrinsic tryptophan fluorescence suggest that it is primarily important for PA-824 binding. Ddn contains five Trp residues (positions 20, 27, 88, 123, and 139), and Ddn protein exhibits an intrinsic fluorescence emission spectrum with a maximum at 330 nm when excited at 280 nm (Figure 7A). In contrast, the NΔ30 Ddn construct showed negligible intrinsic fluorescence, suggesting that residues W20 and W27 are largely responsible for the observed signal. At saturating levels of F_420_, about 60% of tryptophan fluorescence was quenched, whereas PA-824 was able to quench nearly all intrinsic fluorescence (Figure 7A). Similar results were obtained with the MBP-Ddn fusion proteins, providing a direct assay for binding of PA-824 and F_420_ to Ddn, resulting in an apparent K_d_ of 31 μM for PA-824 and 1.0 μM for F_420_ (Figures 7B and 7C).

The proposed binding mode of PA-824 orients the hydrophobic tail toward the expected location of the N terminus of Ddn. Analogues of PA-824 clearly show a preference for monoaryl or biaryl tails (Kim et al., 2009a; Palmer et al., 2010), and suggest the possibility that hydrophobic aromatic residues within the N terminus play a critical role in substrate binding. We therefore created mutations at F16, F17, and W20 to test this hypothesis (Table 2). Single mutations F16L, F16V, or F16A reduced activity of the enzyme toward PA-824 by 60% whereas mutations of F17 or W20 resulted in more variable effects, with loss of 15%–50% of activity as measured in terms of k_cat_/K_M_. Because the apparent flexibility of this region of the protein might allow PA-824 to interact with more than one of these residues, we next studied double and triple mutations. The F16A/F17A mutant still possessed 40% of the activity of the wild-type enzyme, but the triple mutant, F16A/F17A/W20A, had only about 8% activity. Analysis of PA-824 and F_420_ binding by fluorescence spectroscopy showed that both the W20 single mutants and the triple mutant have decreased affinity to PA-824, whereas additional loss of activity in the triple mutant is likely due to a decreased affinity for F_420_. Circular dichroism measurements were used to confirm that none of these mutations caused drastic changes to the overall secondary structure of the protein (Figure S8; Table S5).

## Discussion

Four unique structures of Ddn and the structures of two homologous proteins were determined. The structure of the Ddn protein core with coenzyme F_420_ bound confirms that this protein adopts a split barrel-like fold common among flavin-binding proteins, whereas the N terminus appears to mediate aggregation that prevented study of the full-length WT protein. Details of coenzyme binding and subsequent mutagenesis experiments identified key residues for substrate binding and positioning in the active site. We have postulated a binding mode for PA-824 that involves anchoring the nitroimidazole head group near S78, with Y130 and Y136 participating to position the substrate for reduction (Figure 6). The identification of these residues and the lack of classic catalytic residues in the active site suggest that Ddn functions primarily by precisely orienting PA-824 for efficient direct hydride transfer from F_420_ and potentially through additional transition-state stabilization. Similar mechanisms in which the protein does not directly participate in hydride transfer have been suggested for both an F_420_-utilizing enzyme (Ceh et al., 2009) and a flavin mononucleotide (FMN)-associated enzyme of the split barrel-like fold family (di Salvo et al., 2003) for which ternary donor/acceptor/protein structures are available. This mechanism is also consistent with previous studies of the products of PA-824 reduction that suggested a hydride transfer from F_420_ to the imidazole ring of PA-824 (Singh et al., 2008).

The loss of activity toward PA-824 upon N-terminal truncation of Ddn implies that the N terminus is important for full enzymatic function. The variability of the N termini among homologous proteins of the same protein fold also suggests a role in the specificity of cellular functions. NMR observations and the fact that a defined structure for the N terminus is only observed in crystals of Ddn homologs with F_420_ present support the assignment of the N terminus as a flexible helix near the active site. The proposed substrate orientation places the hydrophobic tail region of PA-824 toward the area occupied by the N terminus of Ddn (that is absent in the structures presented here), which is likely to contribute to substrate affinity as well as solvent exclusion. Extensive mutagenesis studies of the N terminus of the full-length protein (as an MBP fusion to maintain solubility) verified that whereas removing F16, F17, or W20 impaired the enzyme, mutation of all three residues in combination was required to eliminate the majority of enzymatic activity. A flexible N terminus that can interact with the prodrug using any of these three aromatic residues is consistent with the previous synthetic work and quantitative structure-activity relationship modeling using PA-824 and its analogs that have suggested that the tail region of these molecules interacts with one or two hydrophobic aromatic features in Ddn (Kim et al., 2009a, 2009b; Palmer et al., 2010). The importance of these interactions is also supported by recent observations of the effect of stereochemistry of the hydrophobic tail of drug analogs on interactions with Ddn (Gurumurthy et al., 2012).

To our knowledge, the costructures presented here are the first examples of F_420_ bound to this protein fold. Interestingly, all other F_420_-dependent enzymes studied so far, including FGD1, catalyze reactions at the *Si* face of F_420_ (Aufhammer et al., 2004, 2005; Bashiri et al., 2008; Ceh et al., 2009; Johnson and Mukhopadhyay, 2005; Klein et al., 1996; Warkentin et al., 2001). The binding orientation of this coenzyme to Ddn, however, is similar to that seen in split barrel-like FMN-binding proteins (Suto et al., 2000), aligning the *Re* face toward the substrate and implying a possible shared evolutionary origin of Ddn proteins with conventional flavoproteins. This binding mode was supported by the loss of activity of mutants of Y133, Y65, A76, and K79, thought to anchor the coenzyme.

The apo structure of a distantly related protein, Msmeg_3356, an F_420_-dependent aflatoxin reductase from *M. smegmatis*, was recently reported (Taylor et al., 2010). This structure aligns well to the Ddn core (rmsd is 1.3 Å over 109 aligned Cα atoms to Holo-1) and contains an N-terminal helix similar to the homolog structures in our study. Although the physiological functions of these proteins are currently unknown, F_420_-dependent redox biochemistry appears to be widespread in Actinobacteria, and therefore may be important for many pathogenic species.

Ddn is likely to be only one of a class of enzymes that is important for activation of nitroimidazole prodrugs, and a more detailed understanding of the mechanisms of activation and specificity of these enzymes is certain to be important for developing not just PA-824 but other candidates in the anti-TB drug portfolio. Knockout of F_420_ biosynthesis or *fgd1*, but not *Rv3547*, conferred resistance to CGI-17341, a 4-nitroimidazo-oxazole (Figure 1) (Manjunatha et al., 2006a). However, this preference of Ddn for PA-824-like nitroimidazo-oxazines may not be complete, as disruption of the *Rv3547* gene is also reported to convey resistance to OPC-67683 (nitroimidazo-oxazole) (Matsumoto et al., 2006; Gurumurthy et al., 2012). Recent structure-activity relationship modeling also suggests that 4-nitro and 5-nitro compounds have different targets (Kim et al., 2009b). Although these data suggest that developing nitroimidazole compounds will be a complex process, the success of metronidazole and others in this class should be encouraging for the current candidates (Barry et al., 2004). Our efforts only highlight the need for more research into the enzymology and biology of the medical, economic, and humanitarian challenge presented by TB.

## Experimental Procedures

### Materials

Coenzyme F_420_ was purified as described elsewhere (Isabelle et al., 2002; Gurumurthy et al., 2012). The material was characterized by LC-MS and fluorescence emission scanning (mixture of primarily F_420_-4, -5, and -6 at ∼98% purity) and lyophilized for storage. PA-824 was prepared as described (Baker et al., 1997).

### Protein Expression

The Rv3457 coding sequence was cloned into SpeedET and mutations were made using PIPE cloning (Klock et al., 2008). Homolog sequences were synthesized (DNA 2.0) for optimal codon usage in *E. coli* (see Table S2). BL21(DE3) (Invitrogen) cells were grown in LB (Becton Dickinson) or TB (Sigma-Aldrich), moved to 20°C at an OD_600_ of 0.4, induced with IPTG, and harvested the next day. Proteins for SAD phasing were expressed in selenomethionine-containing media. Isotope labeling was done in M9 minimal medium prepared with [^13^C]glucose and/or ^15^NH_4_Cl and 10% Celtone medium (Cambridge Isotope Laboratories) or a mixture of 19 unlabeled and 1 labeled amino acid (Cellitti et al., 2008). Cells were lysed by sonication in 25 mM sodium citrate (pH 6.5), 150 mM KCl, 0.5 mM TCEP. Proteins were purified by Ni-NTA (QIAGEN), TEV digest, a second Ni-NTA column, and a Superdex-75 (GE Healthcare) column in a buffer for the final application. WT and mutant proteins for activity assay were expressed as MBP fusions as previously described (Kim et al., 2009b). WT MBP-Ddn was digested with Precission protease (GE Healthcare) and purified similarly to produce untagged WT Ddn.

### Crystallization and X-Ray Structure Calculations

Proteins were prepared in 10 mM citrate (pH 6.5), 150 mM NaCl, 0.5 mM TCEP at 22, 15–60, or 60 mg/ml for Ddn NΔ30, NΔ33, or NΔ40; or in 20 mM Tris (pH 8), 150 mM NaCl for homologs at 10–15 mg/ml. Crystals were obtained by vapor diffusion using 250 nl sitting drops mixed 1:1 and equilibrated against 50 μl reservoirs (Apo-0/1: 30% PEG6000, 0.1 M MES [pH 6.0] ± 1 M LiCl_2_; Apo-2: 1.6 M [NH_4_]_2_SO_4_, 0.1 M citric acid [pH 4.0]; Holo-1: 1.4 M sodium citrate, 0.1 M HEPES [pH 7.5]; Holo-2: 3.6 M sodium formate, 10% glycerol; nfa33440: 10% 2-methyl-2,4-pentanediol, 0.1 M sodium acetate [pH 5.0]; nfa18080: 1.6 M [NH_4_]_2_SO_4_, 0.1 M HEPES [pH 7.0]) in low-profile Greiner plates (Klock et al., 2008; Santarsiero et al., 2002). Cryoprotectants were added to drops (Apo-0/1/2, Holo-2: 30% sucrose; nfa18080: 30% glycerol). Data were collected at the Advanced Light Source at beamline 5.0.2 for Apo-0/1 crystals (at Se peak wavelength of 0.979 Å) or 5.0.3 for others (0.976 Å fixed wavelength). Data reduction and scaling were performed in HKL2000 (Otwinowski and Minor, 1997). SAD phasing and autobuilding were performed with SOLVE/RESOLVE (Terwilliger, 2000, 2003; Terwilliger and Berendzen, 1999). Molecular replacement was performed with Phaser (McCoy et al., 2007) through the ccp4i interface (CCP4, 1994) using only structures from this study. Iterative model building with Coot (Emsley and Cowtan, 2004) and maximum-likelihood refinement with PHENIX (Adams et al., 2002, 2010) were performed until convergence. Ramachandran plots showed outliers only at K103 (1 of 5 in ASU in Holo-1; 2 of 5 in Holo-2; 2 of 4 at K97 in nfa33440) and Q73 (2 of 4 in nfa33440), both of which are clearly defined in the electron density. In the Holo-1, Holo-2, and nfa33440 maps, some nonspherical density near the F_420_ cofactor was modeled tentatively by waters during refinement, as it could not be satisfactorily modeled as part of the N termini or a small molecule. Structural homologies were calculated using Coot with secondary-structure matching alignment (Krissinel and Henrick, 2004). All protein images were rendered in the PyMOL Molecular Graphics System (Schrödinger, LLC).

### NMR Spectroscopy

Samples were prepared in 90% PBS (pH 7.4) with 10% D_2_O. Isotopically labeled reagents were from Isotec or Cambridge Isotope Laboratories. NMR experiments were acquired at 300 K on a 600 MHz Bruker Avance spectrometer equipped with a 5 mm TXI cryoprobe. NMR experiments and backbone assignment were obtained by standard methods (Mori et al., 1995; Whitehead et al., 1997) as described in Supplemental Information.

### Computational Modeling of PA-824

Docking calculations were performed with software from Schrödinger, LLC using default settings. The Holo-1 crystal structure file was prepared for docking using the Protein Preparation Wizard tool from the FirstDiscovery suite. The potential binding site and optimal grid dimensions were determined with SiteMap. Fully flexible ligand docking was performed with Glide 5.5. The highest-scoring docking solution was further refined by running an LMOD conformational search in Macromodel.

### Activity Assays

Steady-state kinetic parameters for Ddn and its mutants were determined by monitoring the oxidation of F_420_H_2_ by absorbance at 400 nm as previously reported (Kim et al., 2009b; Manjunatha et al., 2006a; Singh et al., 2008). Initial screening of truncation mutants and homologs was performed by monitoring fluorescence at 400 nm/470 nm.

### Ddn Binding to PA-824 and F_420_ Studies

Fluorescence emission spectra of 1 μM Ddn (excitation at 280 nm) were recorded at 310–420 nm. Untagged WT Ddn, WT MBP-Ddn, or mutant MBP-Ddn protein was incubated with varying concentrations of PA-824 or F_420_ for 30 min at room temperature before measurements. Apparent dissociation constant (K_d_) values were calculated by fitting the relative change in intrinsic fluorescence at 330 nm (1 – F/F_0_) versus ligand concentration to a one-site binding (hyperbola) model using nonlinear regression (GraphPad Software). F_0_ is the intrinsic intensity of fluorescence of 1 μM Ddn alone; F is the fluorescence with a given concentration of ligand; corrections were made to subtract ligand-associated fluorescence.

### Circular Dichroism

Circular dichroism (CD) spectra were recorded from 260–195 nm in a Jasco J-715 spectropolarimeter, normalized to absorbance spectra for unpolarized light (McPhie, 2001), and analyzed using the CONTIN program (McPhie, 2008) to determine secondary structure content. See Supplemental Information for additional details.

## Figures and Tables

**Table 1:** Data Collection and Refinement Statistics

Crystal	Apo-0 (NΔ33 Ddn)^a^	Apo-1 (NΔ30 Ddn)	Apo-2 (NΔ33 Ddn)	Holo-1 (NΔ40 Ddn + F_420_-ox)	Holo-2 (NΔ40 Ddn + F_420_-ox)	nfa33440 (+F_420_-ox)	nfa18080 (+F_420_-ox)
PDB ID code		3R5L	3R5P	3R5R	3R5W	3R5Y	3R5Z

**Data Collection**							

Space group	C222_1_	C222_1_	P3_2_21	C2	P2_1_	P4_3_	P2_1_
Cell dimensions							
*a*, *b*, *c* (Å)	67.37, 71.01, 50.74	67.17, 71.04, 50.53	96.18, 96.18, 33.70	146.95, 91.71, 86.50	85.34, 89.42, 127.68	71.96, 71.96, 115.47	39.70, 56.94, 66.00
α, β, γ (°)	90, 90, 90	90, 90, 90	90, 90, 120	90, 119.53, 90	90, 96.29, 90	90, 90, 90	90, 105.52, 90
Resolution (Å)^b^	50–1.75 (1.81–1.75)	50–1.55 (1.61–1.55)	50–1.85 (1.92–1.85)	50–2.10 (2.18–2.10)	50–1.80 (1.86–1.80)	50–1.80 (1.86–1.80)	50–1.50 (1.55–1.50)
R_merge_^b^	8.8 (42.5)	9.8 (62.6)	11.3 (57.2)	11.2 (47.3)	7.3 (72.2)	8.8 (66.5)	8.0 (47.3)
*I*/σ^b^	54.6 (5.7)	27.5 (2.5)	12.4 (1.2)	10.7 (1.9)	22.9 (1.6)	28.2 (1.9)	18.0 (1.6)
Complete (%)^b^	99.1 (91.0)	97.7 (99.5)	97.9 (81.6)	97.9 (98.1)	97.5 (96.3)	100 (100)	93.5 (82.1)
Redundancy^b^	20.6 (18.8)	3.9 (3.3)	5.1 (3.6)	2.4 (2.5)	3.1 (3.2)	5.4 (5.4)	2.3 (1.8)

**Phasing**							

Number found	2						
Figure of merit	0.35						

**Refinement**							

Resolution (Å)		35.52–1.55	41.64–1.85	46.67–2.10	42.17–1.80	45.03–1.80	37.52–1.50
Number of reflections		16,624	14,350	53,828	159,341	51,143	38,957
R_work_/R_free_		18.0/21.1	17.1/20.3	22.7/26.3	17.3/20.6	17.3/21.4	16.6/19.2
Number of atoms							
Protein		942	927	4,465	8,790	4,435	2,261
F_420_ (and other)		12 (MES)	10 (SO_4_^2−^)	265	530	212	111 (SO_4_^2−^)
Water		120	108	438	1,280	486	241
B factors (Å^2^)^c^		24.61	24.63	22.94	31.01	29.37	21.34
Rmsds							
Bond lengths (Å)		1.734	1.747	1.039	1.568	1.131	1.490
Bond angles (°)		0.015	0.018	0.006	0.013	0.008	0.013

a For descriptions of the protein constructs, see also Table S1.

b Values in parentheses are for the highest-resolution shell.

c Average over all atoms.

**Table 2:** In Vitro Activity of MBP-Ddn and Corresponding nfa33440 Mutants toward PA-824

Ddn Mutant^a^	k_cat_^b^ (s^−1^)	K_M_^c^ (μM)	k_cat_/K_M_ (s^−1^μM^−1^)	k_cat_/K_M_ (% WT)	nfa33440 Mutant^a^	k_cat_^b^ (s^−1^)	K_M_^c^ (μM)	k_cat_/K_M_ (s^−1^μM^−1^)	k_cat_/K_M_ (%WT)	Binding by NMR^d^
WT^e^	2.2 (±0.3)	15.5 (±0.06)	0.14 (±0.02)	100	WT^e^	0.33 (±0.08)	21.9 (±8.5)	0.016 (±0.002)	100	
F16L	2.0	30	0.07	47						
F16V	2.2	37	0.06	43						
F16A	2.3	37	0.06	44						
F17L	2.3	18	0.13	93						
F17V	2.5	35	0.07	51						
F17A	2.1	22	0.10	68						
F16/F17A	2.3	45	0.05	37						
F16/F17/W20A^e^	1.2 (±0.01)	104.1 (±4.0)	0.01 (±0.001)	8						
W20F	2.5	20	0.13	93						
W20L	2.6	36	0.07	51						
W20A	2.7	23	0.12	86	W16A	0.2	17	0.012	75	No change
S22A	1.5	15	0.1	71						
S22V	1.3	34	0.04	26						
F41L	1.9	14	0.13	93						
F41A	1.3	26	0.05	36	F35A	<0.1				PA-824 weaker
Q42L	1.2	16	0.08	54						
Y65F^e^	1.1 (±0.04)	10.4 (±1.7)	0.10 (±0.01)	71						
Y65L	<0.1									
Y65A	<0.1				M59A	<0.1				F_420_, PA-824 weaker
A76G	<0.1				V70A	<0.2				
S78A	<0.1				S72A	<0.1				
					S72V	<0.1				PA-824 stronger
K79L	<0.1				Q73A	<0.2				No change
Y130F^e^	0.8 (±0.02)	24.8 (±1.8)	0.03 (±0.004)	21	Y124F	0.28	43	0.007	41	
Y130L	<0.1				Y124L	<0.1				
Y130A	<0.1									
S132V	2.5	52	0.05	34	T126V	0.22	18	0.012	75	No change
S132A	2.1	19	0.11	79						
Y133F	<0.1				Y127F	0.23	85	0.003	17	
Y133L	<0.1				Y127L	<0.1				
Y133A	<0.1									
D135N	2.7	19	0.14	100	E129A	0.43	74	0.006	36	No change
Y136F^e^	2.2 (±0.6)	13.1 (±2.7)	0.16 (±0.02)	114	Y130F	0.31	26	0.012	75	
Y136V	<0.1									
Y136L	<0.1				Y130L	<0.1				
R142A	<0.1				R136A	<0.1				PA-824 weaker
R142L	<0.1				R136Q	<0.1				PA-824 weaker
I144V	1.7	17	0.10	69						
I144A	<0.1				I138A	<0.1				PA-824 weaker
I144G	<0.1									

a See Figure S7 for the locations of all tested mutants.

b For mutants with k_cat_ values <0.2, the K_M_ (and hence k_cat_/K_M_) could not be determined because of the very low reaction rates under the assay conditions.

c K_M_ for PA-824 at saturating [F_420_].

d See Figures S5 and S6 and Tables S3 and S4 for additional information.

e Means with standard errors from triplicate experiments are reported for some proteins.

## Supplemental Information

Document S1. Figures S1–S5 and Tables S2–S5

Table S1. Constructs for Ddn and Homologous Proteins Used in This Study
