# Structure of Ddn, the Deazaflavin-Dependent Nitroreductase from *Mycobacterium tuberculosis* Involved in Bioreductive Activation of PA-824

**DOI:** 10.1016/j.str.2012.12.012

**Published:** 2013-01-08

**Authors:** Susan E. Cellitti, Jennifer Shaffer, David H. Jones, Tathagata Mukherjee, Meera Gurumurthy, Badry Bursulaya, Helena I. Boshoff, Inhee Choi, Amit Nayyar, Yong Sok Lee, Joseph Cherian, Pornwaratt Niyomrattanakit, Thomas Dick, Ujjini H. Manjunatha, Clifton E. Barry, Glen Spraggon, Bernhard H. Geierstanger

**Affiliations:** 1 Genomics Institute of the Novartis Research Foundation, 10675 John Jay Hopkins Drive, San Diego, CA 9212, USA; 2 Tuberculosis Research Section, National Institute of Allergy and Infectious Diseases, National Institutes of Health, Bethesda, MD 20892, USA; 3 Novartis Institute for Tropical Diseases, 138670 Singapore; 4 Center for Molecular Modeling, Center for Information Technology, National Institutes of Health, Bethesda, MD 20892, USA

## Main Text

(Structure *20*, 101–112, January 11, 2012)

The original article unfortunately has an author error in the units for *k*_*cat*_ and *k*_*cat*_*/K*_*m*_ on page 108. In Table 2, row 2, all *k*_*cat*_ and *k*_*cat*_*/K*_*m*_ units should be “min^−1^” and “min^−1^μM^−1^,” respectively, rather than be “s^−1^” and “s^−1^μM^−1^.” The scientific interpretations remain the same, only the units change. Our conclusions about the active site structure based on the kinetic analysis of the mutants do not change.

