# Supplementary material for: Structure of Ddn, the Deazaflavin-Dependent Nitroreductase from Mycobacterium tuberculosis Involved in Bioreductive Activation of PA-824
Source: Structure. 2012 Jan 11;20(1-2):101–12. doi: 10.1016/j.str.2011.11.001 (PMC3267046; doi:10.1016/j.str.2011.11.001)
Supplement: Document S1. Figures S1–S5 and Tables S2–S5 [file mmc1.pdf]

Structure, Volume *20*

## Supplemental Information

# Structure of Ddn, the Deazaflavin-Dependent Nitroreductase from *Mycobacterium tuberculosis* Involved in Bioreductive Activation of PA-824

**Susan E. Cellitti, Jennifer Shaffer, David H. Jones, Tathagata Mukherjee, Meera Gurumurthy, Badry Bursulaya, Helena I. Boshoff, Inhee Choi, Amit Nayyar, Yong Sok Lee, Joseph Cherian, Pornwaratt Niyomrattanakit, Thomas Dick, Ujjini H. Manjunatha, Clifton E. Barry III, Glen Spraggon, and Bernhard H. Geierstanger**

**Inventory of Supplemental Information**

**Supplementary Table S1, Related to Table 1**
**Constructs for Ddn and homologous proteins used in this study**
**Supplementary Figure S1, Related to Figure 2**
**Characterization of WT and truncated Ddn**
**Supplementary Figure S2, Related to Figure 2**
**Predicted N-terminal helix of Ddn**
**Supplementary Figure S3, Related to Figure 2**
**Comparison of NΔ30 and helix mutants**
**Supplementary Table S2, Related to Figure 3**
**Partial NMR chemical shift assignments for NΔ30 with and without $F_{420}$**
**Supplementary Figure S4, Related to Figure 5**
**NMR characterization of nfa33440**
**Supplementary Figure S5, Related to Table 2**
**NMR characterization of nfa33440 mutants**
**Supplementary Table S3, Related to Table 2**
**Half-height linewidth[1] of the 0.45 ppm methyl peak of nfa33440 mutants at different protein to $F_{420}$ coenzyme ratios**
**Supplementary Table S4, Related to Table 2**
**PA-824 chemical shift difference and T1ρ intensity loss in the presence of nfa33440 mutants**
**Supplementary Figure S6, Related to Table 2**
**PA-824 titration of nfa33440 S72V**
**Supplementary Figure S7, Related to Table 2**
**Cartoon modelss of Ddn (Holo-1) and nfa33440 showing positions of residues mutated for functional studies**
**Supplementary Figure S8, Related to Figure 7**
**Circular dichroism of the wild type DDn, maltose binding protein, F16A-F17A-W20A-Ddn and F16A-F17A-Ddn is represented as a plot of g-factor vs. wavelength**
**Supplementary Table S5, Related to Figure 7**
**Relative percentage of secondary structure components as derived from circular dichroism measurements of the wild type DDn, maltose binding protein, F16A-F17A-W20A-Ddn and F16A-F17A-Ddn**

**Supplementary Figure S1, Related to Figure 2**

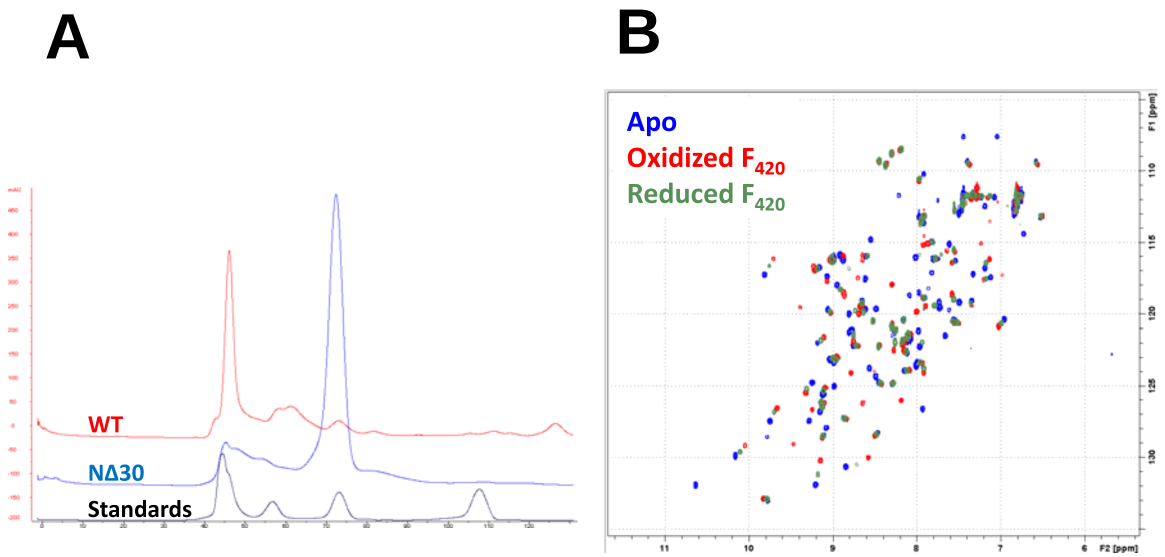


**Figure S1. Characterization of WT and truncated Ddn**

(A) Preparative size exclusion chromatography illustrates the difference in behavior of full-length (red/top) and truncated (blue/middle) Ddn. While the full-length protein elutes near the void volume as a soluble aggregate, the NΔ30 truncation mutant elutes as expected for a monomeric protein. BioRad standards (black) are included for comparison. Both proteins were expressed from SpeedET. NΔ30 and standards were run in PBS; full-length was run in 25 mM citrate pH 6.5, 150 mM NaCl.

(B) NMR spectra of NΔ30 with and without coenzyme $F_{420}$. Dispersed peaks in these $^{1}H$-$^{15}N$-HSQC spectra suggest a well folded protein with a distinct three dimensional structure. Shifted peaks upon addition of coenzyme indicate tight binding. The apo spectrum (blue) includes 113 of an expected 114 peaks (including N-terminal Gly from TEV site). The oxidized (red) and reduced (green) coenzyme spectra contain 100 and 84 peaks respectively. Partial assignments for the apo- and oxidized $F_{420}$ spectra are presented in Supplementary Table 2.

**Supplementary Figure S2, Related to Figure 2**

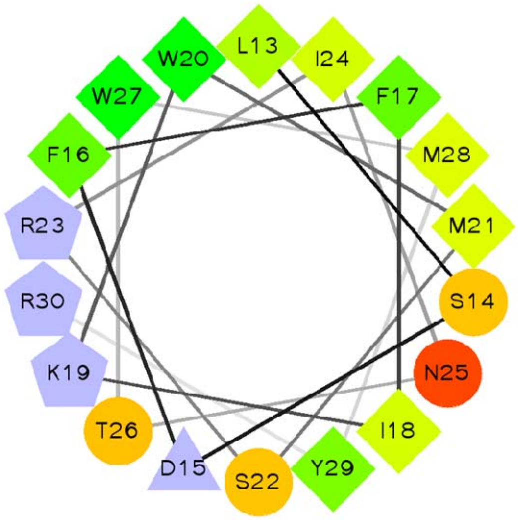


**Figure S2. Predicted N-terminal helix of Ddn**

Residues 13-28 are predicted to form a helix with a strongly hydrophobic face, as shown in this projection(Armstrong and Zidovetzki). This may be the cause of the aggregation of the full-length protein. It is unclear if Ddn is membrane-associated *in vivo*, but it has been identified in detergent extractions of *M. tuberculosis* as described in the main text(Sinha et al., 2002; Sinha et al., 2005). The N-termini of Ddn and similar predicted split barrel-like proteins vary greatly and may be important for their native functions.

**Supplementary Figure S3, Related to Figure 2**

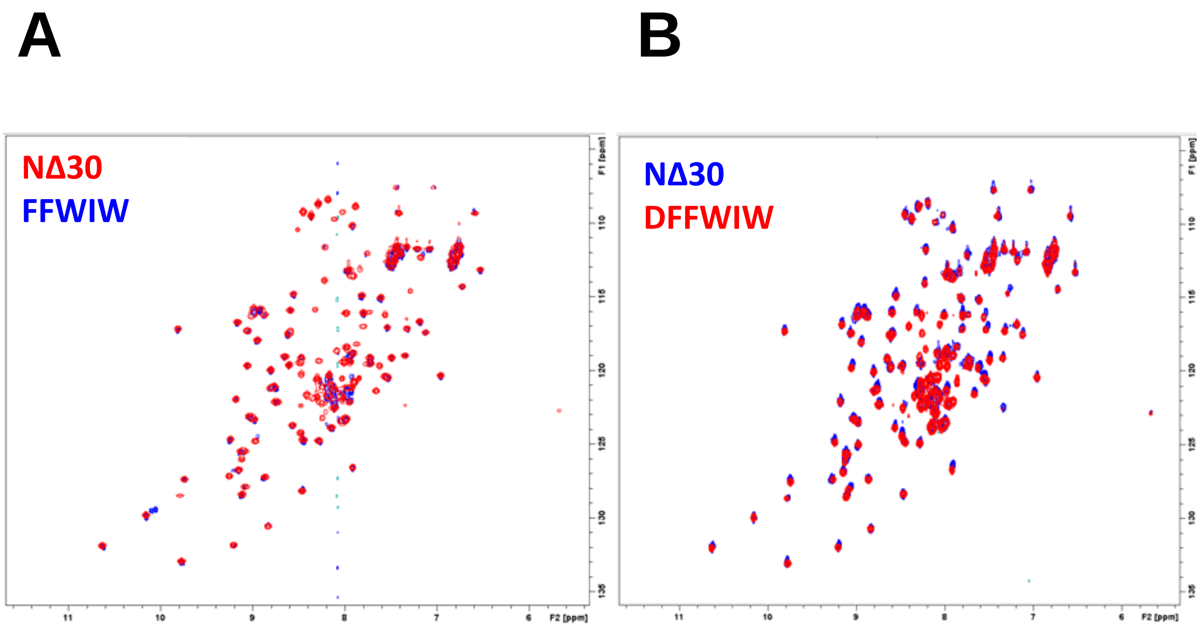


**Figure S3. Comparison of NΔ30 and helix mutants**

Initially, mutations were made to the predicted N-terminal helix in order to remodel the hydrophobic face and prevent aggregation. Like the N-terminal truncations, these mutants behaved as monomeric proteins as determined by analytical size exclusion chromatography (see Table S1).

(A) The $^{1}H$-$^{15}N$-HSQC spectra of these mutants (F16A-F17D-W20D-I24A-W27D full-length Ddn shown here in blue) are very similar to that of NΔ30 (in red). This suggests that the N-termini of the mutants undergo conformational exchange.

(B) Constructs were made to introduce up to three salt bridges into the putative N-terminal helix in order to stabilize its structure. The $^{1}H$-$^{15}N$-HSQC spectra of these full-length Ddn mutants (D15E-F16A-F17D-W20D-I24A-W27D shown here) are very similar to that of NΔ30, except that there are approximately 18 additional NH peaks (out of 25 possible). This suggests a change in the kinetics of the conformational exchange process. However, the lack of shifts (beyond error in linewidths) among the shared peaks suggests that the N-terminus is not interacting with the core of the protein in the mutants.

Cellitti et al.

Structure of Ddn, the Deazaflavin-dependent nitroreductase from *Mycobacterium tuberculosis* involved in bioreductive activation of PA-824

**Supplementary Table S2, Related to Figure 3**

**Table S2. Partial NMR chemical shift assignments for NΔ30 with and without $F_{420}$**

| | Apo | | with $F_{420}$ [1] | |
|---|---|---|---|---|
| Residue | N (ppm) | HN (ppm) | N (ppm) | HN (ppm) |
| N32 | 120.541 | 8.518 | 120.622 | 8.506 |
| D33 | 120.883 | 8.302 | 120.795 | 8.29 |
| G34 | 108.815 | 8.302 | 108.807 | 8.292 |
| E35 | 120.424 | 8.151 | 120.469 | 8.15 |
| G36 | 109.654 | 8.376 | 109.592 | 8.359 |
| L37 | 121.455 | 8.103 | 121.406 | 8.075 |
| G38 | 109.342 | 8.45 | 109.389 | 8.443 |
| G39 | 108.52 | 8.185 | 108.558 | 8.191 |
| T40 | 113.247 | 7.954 | 113.358 | 7.949 |
| F41 | 121.997 | 8.176 | 122.035 | 8.167 |
| Q42 | 121.455 | 8.103 | 121.892 | 8.14 |
| K43 | 122.457 | 8.242 | 122.565 | 8.265 |
| I44 | 123.723 | 8.043 | 124.119 | 7.916 |
| V46 | 116.069 | 8.003 | 115.187 | 7.912 |
| A47 | 125.545 | 9.109 | 126.718 | 9.247 |
| L48 | 119.607 | 8.479 | 117.917 | 8.292 |
| L49 | 130.664 | 8.838 | 129.968 | 8.563 |
| T50 | 127.455 | 9.742 | 126.572 | 9.659 |
| T51 | 116.14 | 8.986 | 115.949 | 8.866 |
| T52 | 115.916 | 8.907 | 117.721 | 9.063 |
| G53 | 114.885 | 8.554 | 116.594 | 9.222 |
| R54 | 126.677 | 7.911 | 126.004 | 8.177 |
| K55 | 118.293 | 8.57 | 119.584 | 9.388 |
| T56 | 114.935 | 8.549 | 111.517 | 9.939 |
| G57 | 110.231 | 7.912 | 110.698 | 7.967 |
| Q58 | 119.208 | 7.723 | 119.856 | 7.996 |
| R60 | 124.768 | 9.239 | 125.501 | 9.315 |
| V61 | 124.334 | 8.484 | 126.222 | 8.64 |
| N62 | 124.984 | 8.977 | | |
| L64 | 127.398 | 9.27 | | |
| Y65 | 121.496 | 7.663 | | |
| F66 | 113.639 | 7.908 | | |
| L67 | 116.244 | 8.871 | 117.447 | 8.694 |
| R68 | 121.148 | 8.764 | 121.884 | 8.743 |
| D69 | 123.367 | 7.981 | 123.4 | 7.922 |
| G70 | 116.14 | 8.986 | 116.203 | 9.03 |
| G71 | 116.377 | 9.023 | 116.24 | 8.972 |
| R72 | 116.153 | 7.792 | 116.139 | 7.768 |
| V73 | 122.177 | 8.745 | 122.243 | 8.695 |
| I74 | 131.897 | 9.201 | 130.225 | 9.142 |
| V75 | 117.375 | 9.056 | | |
| A76 | 121.683 | 8.069 | | |
| A77 | 119.301 | 8.016 | | |
| E83 | 118.509 | 7.981 | | |
| K84 | 121.756 | 8.046 | | |
| N85 | 117.474 | 8.563 | | |
| M87 | 122.169 | 8.699 | | |
| W88 | 115.119 | 7.603 | | |
| Y89 | 122.769 | 5.667 | 121.985 | 6.036 |
| L90 | 117.212 | 7.318 | 117.282 | 6.965 |
| N91 | 117.542 | 8.607 | 118.777 | 8.822 |
| L92 | 119.53 | 7.719 | 119.458 | 7.888 |
| K93 | 120.47 | 7.552 | 120.628 | 7.552 |
| A94 | 120.391 | 6.951 | 120.874 | 7.017 |
| N95 | 114.945 | 7.808 | 115.078 | 7.862 |
| K97 | 119.204 | 7.487 | 118.661 | 7.573 |
| V98 | 119.101 | 8.645 | 119.462 | 8.654 |
| Q99 | 118.894 | 7.905 | 118.871 | 7.919 |
| V100 | 123.158 | 9.029 | 123.03 | 8.947 |
| Q101 | 127.278 | 8.859 | 127.338 | 8.839 |
| I102 | 128.32 | 8.462 | 128.483 | 8.491 |
| K103 | 126.059 | 9.124 | 126.265 | 9.141 |
| K104 | 121.281 | 8.256 | 121.051 | 8.232 |
| E105 | 122.638 | 8.11 | 122.546 | 8.137 |
| V106 | 124.84 | 8.277 | 124.796 | 8.277 |
| L107 | 128.469 | 9.108 | 128.612 | 9.119 |
| D108 | 124.763 | 8.441 | 124.867 | 8.415 |
| L109 | 123.939 | 8.137 | 123.885 | 8.083 |
| T110 | 116.77 | 9.153 | 116.92 | 9.203 |
| A111 | 132.978 | 9.769 | 132.901 | 9.82 |
| R112 | 117.976 | 8.941 | 118.511 | 8.862 |
| D113 | 120.002 | 8.8 | 119.462 | 8.654 |
| A114 | 123.756 | 8.558 | 124.09 | 8.777 |
| T115 | 117.226 | 9.805 | 116.131 | 9.692 |
| D116 | 122.009 | 9.174 | 121.604 | 9.096 |
| E117 | 119.693 | 8.6 | 120.027 | 8.693 |
| E118 | 120.47 | 7.552 | 120.658 | 7.505 |
| R119 | 119.74 | 9.054 | 119.963 | 9.023 |
| A120 | 117.463 | 7.121 | 117.629 | 7.199 |
| E121 | 116.74 | 7.179 | 116.182 | 7.12 |

[1]Samples were titrated with $F_{420}$ to full occupancy before data acquisition for assignments.

**Supplementary Table S2, Related to Figure 3**

## Table S2 (cont.)

| | Apo | | with $F_{420}$[1] | |
|---|---|---|---|---|
| Residue | N (ppm) | HN (ppm) | N (ppm) | HN (ppm) |
| Y122 | 115.978 | 8.595 | | |
| W123 | 121.243 | 8.805 | | |
| Q125 | 114.374 | 6.719 | | |
| L126 | 122.24 | 7.953 | | |
| Y136 | 119.599 | 7.76 | | |
| Q137 | 119.419 | 8.276 | | |
| S138 | 113.16 | 7.834 | | |
| D141 | 120.699 | 7.342 | | |
| R142 | 121.851 | 8.112 | | |
| T143 | 111.755 | 8.202 | | |
| I144 | 122.465 | 7.352 | | |
| I146 | 121.225 | 7.973 | | |
| V147 | 127.931 | 9.076 | | |
| V148 | 126.807 | 9.144 | | |
| C149 | 125.545 | 9.109 | 125.142 | 9.084 |
| E150 | 123.352 | 8.981 | 123.03 | 8.947 |

**Supplementary Figure S4, Related to Figure 5**

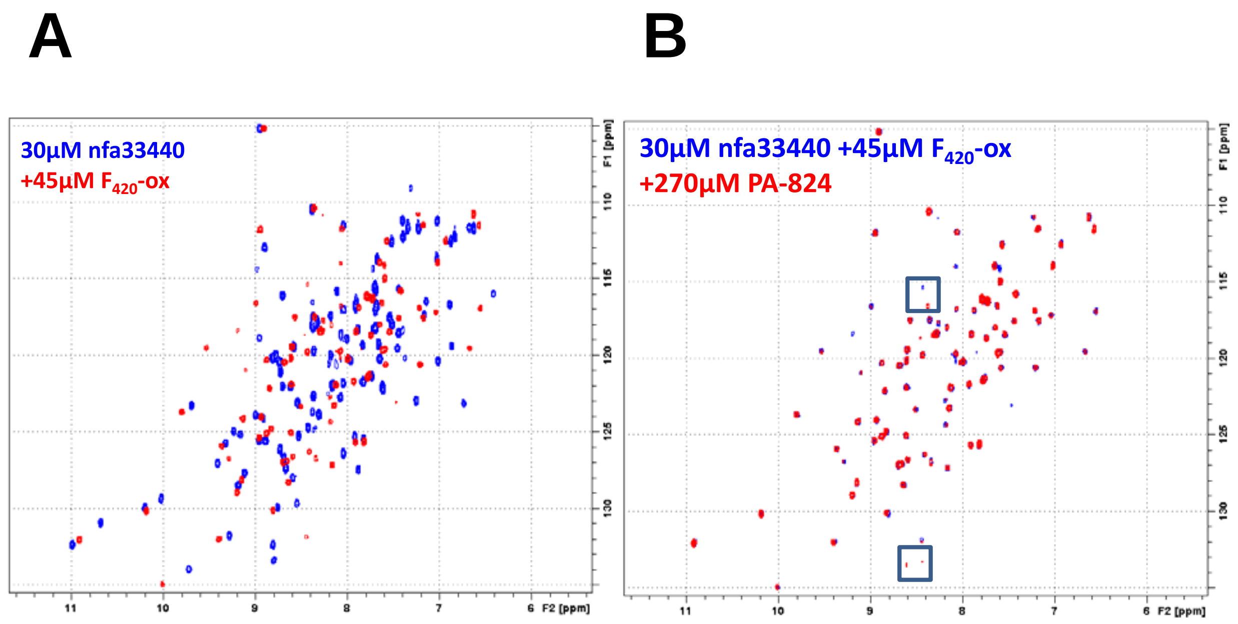


**Figure S4. NMR characterization of nfa33440**

(A) The $^{1}$H-$^{15}$N-HSQC spectrum of apo-nfa33440 includes 107 of 135 expected peaks (blue). Although no assignments have been made, the number of peaks is suggestive of a structured protein core with the N-terminus undergoing conformational exchange in the intermediate time regime. Robust shifts upon addition of $F_{420}$ confirm tight binding of the coenzyme. In the presence of $F_{420}$ (red), there are approximately 90 peaks observed. This is similar to the case for Ddn NΔ30 in which parts of the active site are no longer visible upon binding to coenzyme.

(B) Addition of PA-824 does not change the number of peaks observed (red), but there are chemical shift perturbations of some of the weaker peaks. This further supports the hypothesis that it is the active site residues that are undergoing exchange and that binding of the substrates of the enzyme affects these dynamic processes.

**Supplementary Figure S5, Related to Table 2**

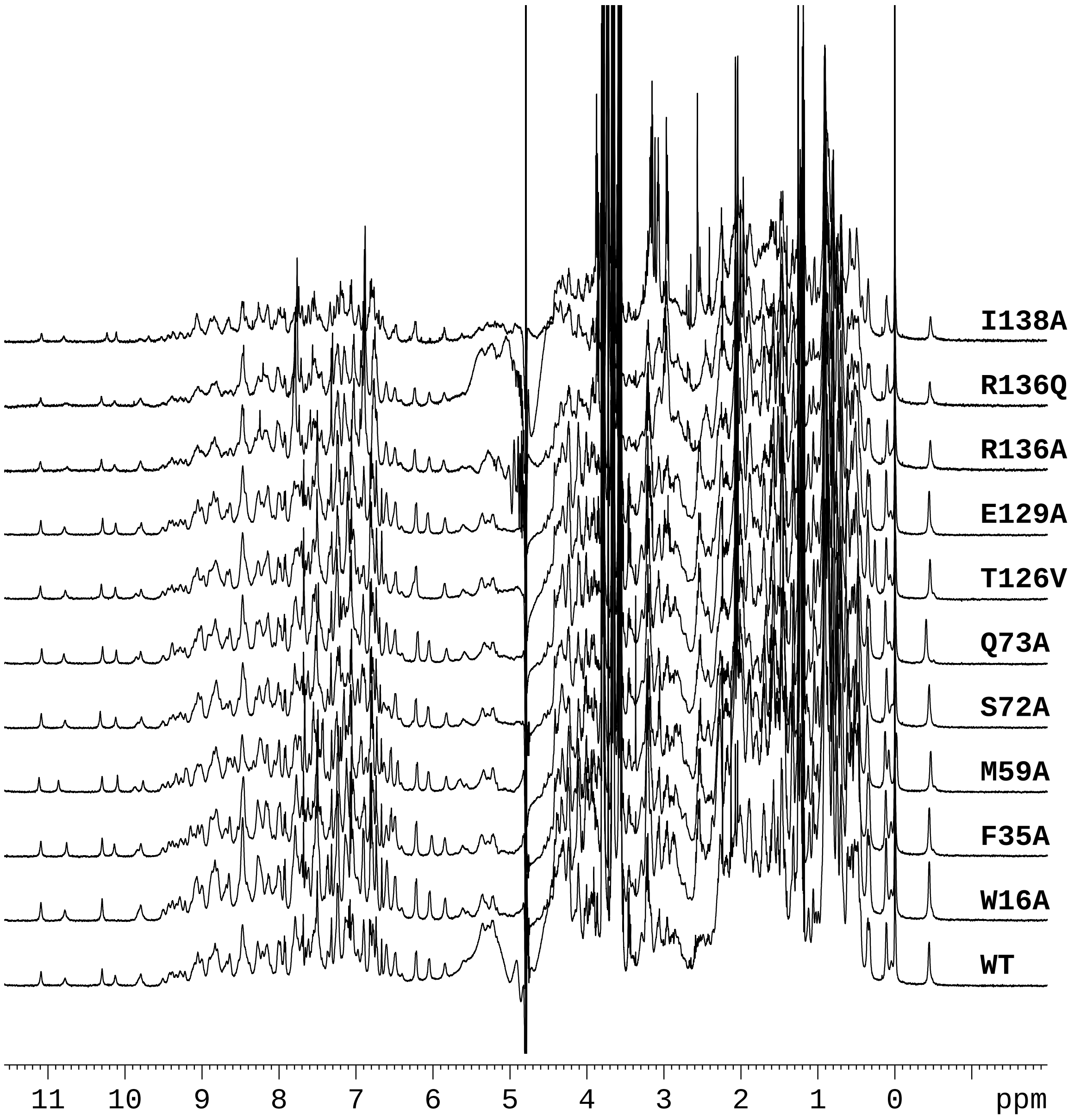


**Figure S5. NMR characterization of nfa33440 mutants**

Proton spectra of the indicated mutants are shown. All samples were acquired in PBS using excitation sculpting for water suppression(Hwang and Shaka, 1995). The appearance of the spectra for all the mutants is very similar to wild-type protein and confirms they are well-folded with similar structures. The peaks for M59A have the largest chemical shift changes compared to wild-type and suggest this mutation may have had the most significant impact on the structure. The mutants W16A and T126A are missing peaks at 10.13 and 6.05 ppm respectively, probably corresponding to the amino acid amide protons.

**Supplementary Table S3, Related to Table 2**

**Table S3. Half-height linewidth[1] of the 0.45 ppm methyl peak of nfa33440 mutants at different protein to $F_{420}$ coenzyme ratios**

| | | Width of upfield methyl line (Hz) | | |
|---|---|---|---|---|
| **Sample** | **Apo** | **1.4:1 $F_{420}$** | **2:1 $F_{420}$** | **3:1 $F_{420}$** |
| **I138A** | 23 | 37 | 20 | |
| **R136Q** | 24 | 37 | 24 | |
| **F136A** | 21 | 40 | 25 | |
| **E129A** | 16 | 37 | 19 | |
| **T126V** | 16 | 46 | 22 | |
| **Q73A** | 15 | 18 | 15 | |
| **S72V** | 18 | 26 | | |
| **M59A** | 17 | 117 | 85 | 39 |
| **F35A** | 18 | 50 | 39 | |
| **W16A** | 17 | 46 | 26 | |
| **WT** | 17 | 42 | 18 | |

[1]The linewidth is sensitive to $F_{420}$ occupancy and increases when the protein is a mixture of free and bound - presumably due to exchange between apo and complex. Consequently, the concentration of coenzyme required to achieve a linewidth comparable to apo protein is a useful indication of the concentration required for 100% occupancy and thus the affinity. The results indicate that the only mutant with compromised coenzyme binding is M59A.

**Table S4. PA-824 chemical shift difference and T1ρ intensity loss in the presence of nfa33440 mutants**

| Sample[1] | δ-δapo [Hz][2] PA-824 Peak (ppm) | | | | | T1ρ intensity loss [%][3] PA-824 Peak (ppm) | | | | |
|---|---|---|---|---|---|---|---|---|---|---|
| | 7.775 | 7.459 | 7.444 | 7.339 | 7.325 | 7.775 | 7.459 | 7.444 | 7.339 | 7.325 |
| S72V[5] | no signals for PA-824 (DMSO peak only) | | | | | no signals for PA-824 | | | | |
| M59A[4] | -2.7 | -1.9 | -1.9 | -2.6 | -1.7 | 14 | 14 | 14 | 25 | 20 |
| F35A[4] | -1.6 | -0.4 | -0.4 | -1.3 | -0.2 | 14 | 20 | 16 | 40 | 40 |
| I138A[4] | -2.2 | -2.5 | -2.5 | -2.5 | -2.3 | 13 | 20 | 18 | 18 | 25 |
| R136Q[4] | -2.0 | -2.0 | -2.0 | -2.6 | -2.0 | 22 | 22 | 22 | 35 | 35 |
| R136A[4] | -2.2 | -2.5 | -2.5 | -2.5 | -2.3 | 13 | 20 | 18 | 18 | 25 |
| Q73A | -32.6 | -4.0 | -3.9 | -4.1 | -4.1 | 71 | 35 | 35 | 44 | 44 |
| W16A | -37.3 | 2.3 | 2.2 | 3.8 | -4.1 | 72 | 32 | 32 | 58 | 58 |
| T126V | -21.1 | -4.4 | -4.4 | -4.2 | -3.8 | 71 | 23 | 23 | 56 | 56 |
| E129A | -30.8 | -3.1 | -3.1 | -2.7 | -3.1 | 82 | 34 | 34 | 55 | 55 |
| WT | -17.3 | -3.6 | -3.5 | -3.0 | -3.4 | 78 | 25 | 25 | 57 | 57 |

[1]Samples were prepared with the listed mutant at a protein-to-$F_{420}$ ratio of 1:1.4 to 1:2 and a protein-to-PA-824 ratio of 1:0.6.

[2]The chemical shift changes in Hz were measured for five peaks of PA-824 by subtracting the chemical shift in the presence of protein from those measured in buffer alone. For a two state system undergoing fast exchange ($k >> 2\pi\Delta\delta$), the chemical shift should reflect the average of the two populations(Wuthrich, 1986). Assuming that the chemical shift of the bound form is similar for all mutants, a larger chemical shift change reflects a greater bound fraction and provides an estimate of the relative affinity.

[3]T1ρ data were also acquired to provide additional confirmation. In this experiment, spectra were acquired using a CPMG sequence with a short (10 ms) and long (200 ms) mix time, and the percent intensity loss was determined (Intensity loss = 1-($Intensity_{200ms}$/$Intensity_{10ms}$). The intensity loss is related to the fraction bound(Dalvit, 2009).

[4]The grey shaded mutants appear to have altered PA-824 binding affinity based on T1ρ and chemical shift difference data.

[5]For S72V there is no indication of free compound peaks despite the appearance of a DMSO peak (compound was added from a DMSO stock). These data suggest that compound binding is in the intermediate exchange regime ($k \approx 2\pi\Delta\delta$) resulting in significant line broadening. This is unlike all the other mutants and wild-type where PA-824 is clearly in a fast-exchange regime and suggests that this mutant may have increased affinity for the prodrug. This may be due to a change in the binding pose or a stronger hydrophobic interaction with the substrate but does not represent a more efficient orientation for reduction based on the activity measurements.

**Figure S6, Related to Table 2**

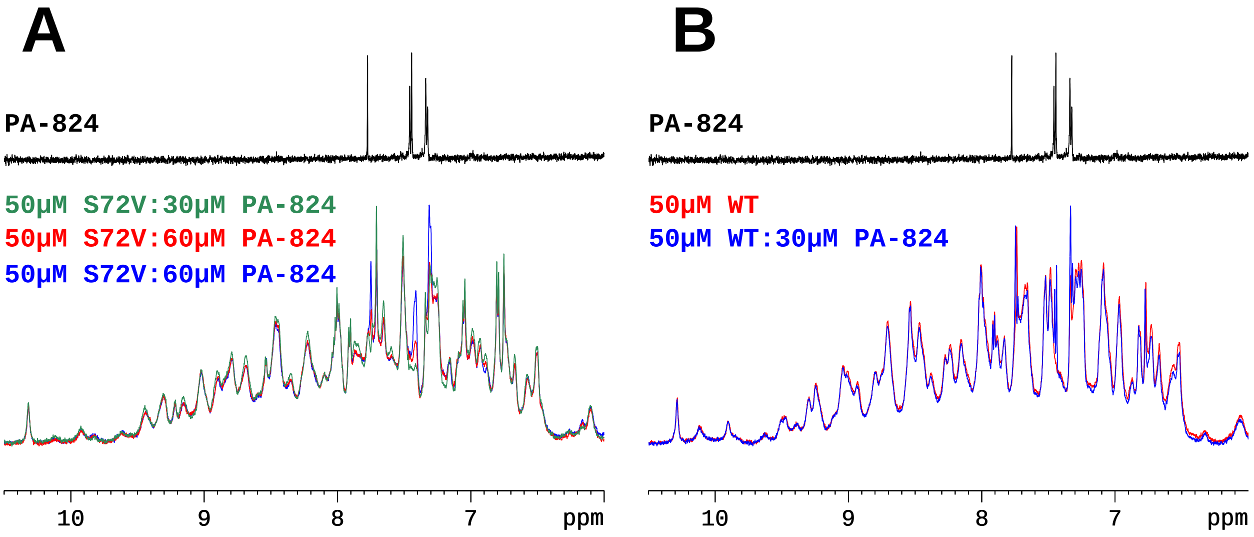


**Figure S6. PA-824 titration of nfa33440 S72V**

(A) Overlaid proton spectra of 50 µM S72V with 30, 60, and 90 µM PA-824 in green, red, and blue respectively.

(B) Overlaid spectra for 50 µM wild-type protein alone (red) and upon the addition of 30 µM PA-824 (blue). In each panel the spectrum for PA-824 is offset in order to readily identify compound peaks. Sharp peaks corresponding to PA-824 were observed for wild-type nfa33440 at a protein-to-drug ratio of 1:0.6. Compound peaks were not observed for the S72V mutant until much higher concentrations (a protein-to-drug ratio of 1:1.8) and were very broad. These data suggest that the S72V mutant has higher PA-824 affinity.

**Supplementary Figure S7, Related to Table 2**

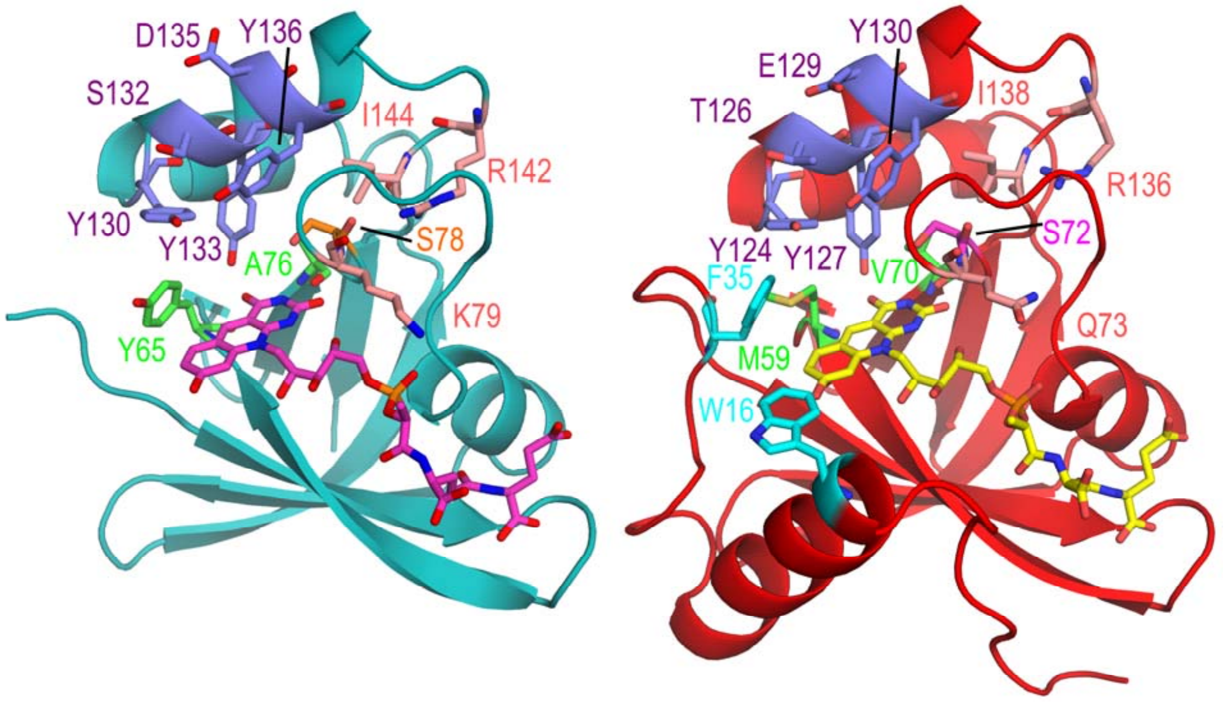


**Figure S7. Cartoon of Ddn (Holo-1) and nfa33440 showing positions of residues mutated for functional studies.** Residues tested that are also present in the structure are shown as sticks and colored in groups related to the presentation in Table 2.

**Supplementary Figure S8, Related to Figure 7**

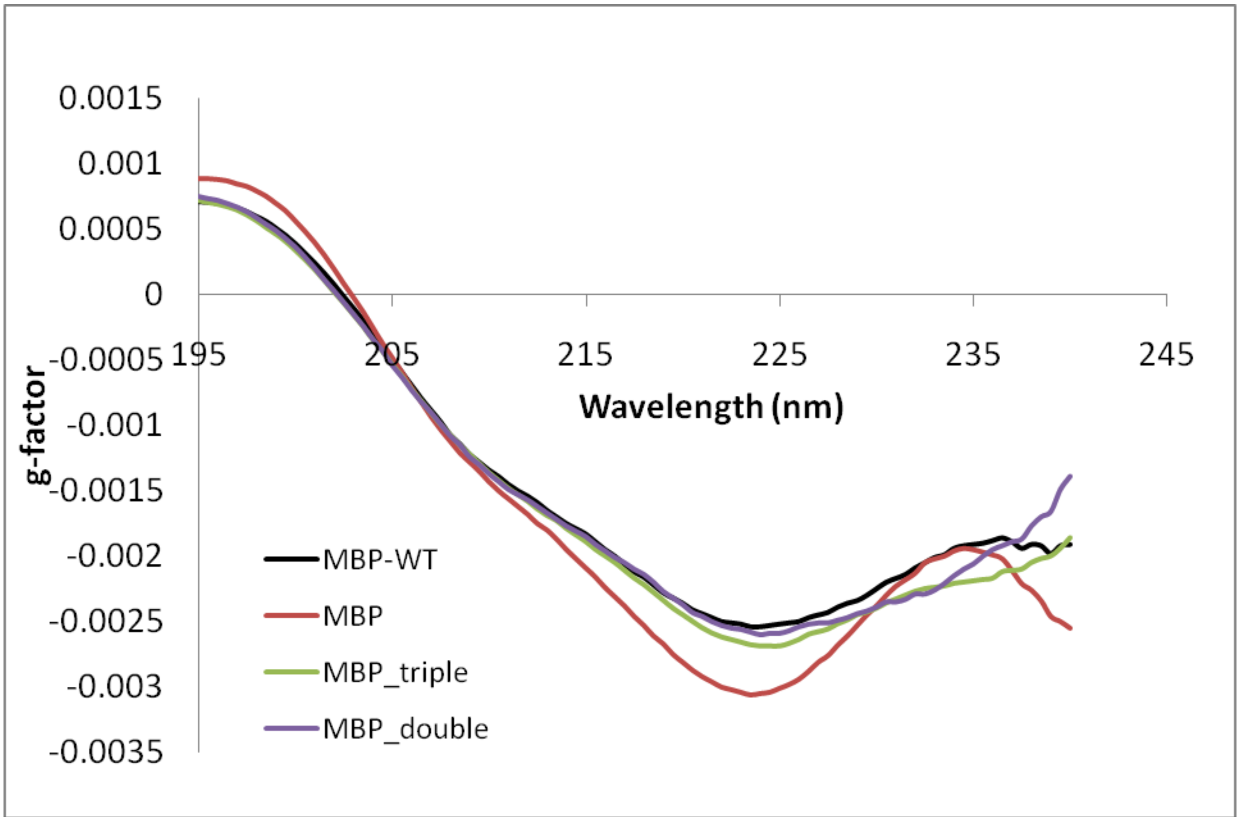


**Figure S8. Circular dichroism of the wild type Ddn (MPB-WT, black), maltose binding protein (MBP, red), F16A-F17A-W20A-Ddn (MBP-triple, green) and F16A-F17A-Ddn (MBP-double, blue) is represented as a plot of g-factor vs. wavelength**

Structure of Ddn, the Deazaflavin-dependent nitroreductase from *Mycobacterium tuberculosis* involved in bioreductive activation of PA-824

**Supplementary Table S5, Related to Figure 7**

**Table S5. Relative percentage of secondary structure components as derived from circular dichroism measurements of the wild type DDn (MPB-WT, black), maltose binding protein (MBP, red), F16A-F17A-W20A-Ddn (MBP-triple, green) and F16A-F17A-Ddn (MBP-double, blue)**

| | MBP-WT | MBP | MBP-Triple | MBP-Double |
|---|---|---|---|---|
| helix | 26+/1.4% | 33+/-2.5% | 26+/-1.2% | 28+/-1.4% |
| sheet | 30+/-1.5 | 28+/-2.4 | 31+/-1.2 | 30+/-1.3 |
| turn | 11+/-1.1 | 12+/-1.9 | 11+/-1.0 | 12+/-1.1 |
| rem. | 33+/-1.1 | 28+/-1.9 | 31+/-0.9 | 30+/-1.1 |

## REFERENCES


Armstrong, D., and Zidovetzki, R. http://rzlabucredu/scripts/wheel/wheelcgi.

Dalvit, C. (2009). NMR methods in fragment screening: theory and a comparison with other biophysical techniques. Drug Discov Today *14*, 1051-1057.

Hwang, T.L., and Shaka, A.J. (1995). Water suppression that works - Excitation sculpting using arbitrary wave-forms and pulsed-field gradients. J Magn Reson Ser A *112*, 275-279.

Sinha, S., Arora, S., Kosalai, K., Namane, A., Pym, A.S., and Cole, S.T. (2002). Proteome analysis of the plasma membrane of *Mycobacterium tuberculosis*. Comp Funct Genomics *3*, 470-483.

Sinha, S., Kosalai, K., Arora, S., Namane, A., Sharma, P., Gaikwad, A.N., Brodin, P., and Cole, S.T. (2005). Immunogenic membrane-associated proteins of *Mycobacterium tuberculosis* revealed by proteomics. Microbiology *151*, 2411-2419.

Wuthrich, K. (1986). NMR of proteins and nucleic acids (New York, NY, Wiley-Interscience).
